# Distribuição Espacial de Pacientes na Região Metropolitana de São Paulo Atendidos em um Hospital Público Terciário de Referência Cardiovascular Segundo Doenças dos Sistemas Circulatório, Respiratório, Endócrino e Neoplásico

**DOI:** 10.36660/abc.20260178

**Published:** 2026-06-26

**Authors:** Carlos Lederman, Sara Lopes de Moraes, Antonio Carlos Pedroso de Lima, Lucia Pereira Barroso, Lilian Cristina Correia Morais, Magaly de Losso Perdigão, Rosa Maria Vieira de Freitas, Monica La Porte Teixeira, Bernadette Cunha Waldvogel, Marco Antonio Gutierrez, Ligia Vizeu Barrozo, Alfredo José Mansur

**Affiliations:** 1 Faculdade de Medicina Universidade de São Paulo São Paulo SP Brasil Faculdade de Medicina da Universidade de São Paulo (FMUSP), São Paulo, SP – Brasil; 2 Universidade de São Paulo Faculdade de Filosofia, Letras e Ciências Humanas São Paulo SP Brasil Universidade de São Paulo – Faculdade de Filosofia, Letras e Ciências Humanas, São Paulo, SP – Brasil; 3 Universidade de São Paulo Instituto de Matemática e Estatística São Paulo SP Brasil Universidade de São Paulo – Instituto de Matemática e Estatística, São Paulo, SP – Brasil; 4 Fundação Sistema Estadual de Análise de Dados - Projeções Populacionais São Paulo SP Brasil Fundação Sistema Estadual de Análise de Dados - Projeções Populacionais, São Paulo, SP – Brasil; 5 Hospital das Clínicas Faculdade de Medicina Universidade de São Paulo São Paulo SP Brasil Instituto do Coração do Hospital das Clínicas da Faculdade de Medicina da Universidade de São Paulo, São Paulo, SP – Brasil

**Keywords:** Classificação Internacional de Doenças, Comorbidade, Causas de Morte, Mortalidade, Doenças Não Transmissíveis

## Abstract

**Fundamento:**

A distribuição espacial de pacientes segundo características clínicas, utilizando técnicas de geoprocessamento, permite identificar aglomerados de doenças em uma população estudada. Essa abordagem contribui para o desenvolvimento de intervenções direcionadas a grupos específicos de risco.

**Objetivos:**

Investigar aglomerados geográficos de doenças de interesse entre pacientes atendidos em um hospital público terciário e caracterizar sua distribuição espacial na região metropolitana de São Paulo.

**Métodos:**

Com base nos resultados de um estudo prévio, foram definidos dois grupos de doenças: um representando as principais doenças identificadas na primeira consulta hospitalar e outro representando as principais causas básicas de morte. Uma descrição detalhada é apresentada no material suplementar online. A densidade das doenças foi calculada dividindo-se a frequência de casos em cada grupo de doenças pela população de cada cidade/distrito e multiplicando-se o resultado por 100.000 para melhorar a visualização dos dados. Posteriormente, os dados espaciais foram analisados e mapeados para ilustrar os padrões espaciais e as relações identificadas no estudo.

**Resultados:**

Osasco e Taboão da Serra apresentaram a maior densidade de causas básicas de morte relacionadas a doenças do sistema circulatório. Além disso, 12 cidades/distritos localizados nas fronteiras da área de estudo não apresentaram registro de doenças neoplásicas.

**Conclusões:**

Vargem Grande Paulista apresentou a maior densidade de casos de neoplasias como causas básicas de morte e comorbidades. Destaca-se que Embu das Artes, município reconhecido por suas extensas áreas verdes, apresentou a maior densidade de doenças do sistema respiratório.

## Graphical abstract

**Figura Central:**
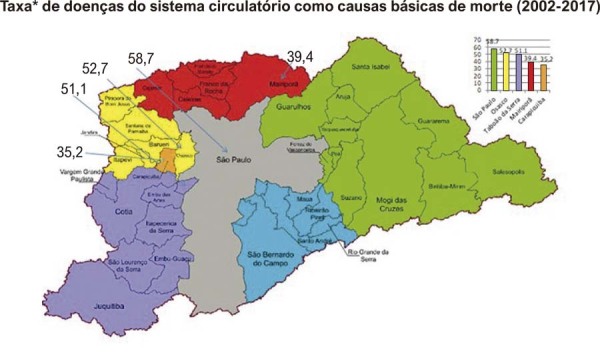
Distribuição Espacial de Pacientes na Região Metropolitana de São Paulo Atendidos em um Hospital Público Terciário de Referência Cardiovascular Segundo Doenças dos Sistemas Circulatório, Respiratório, Endócrino e Neoplásico

*Taxa = frequência de casos ÷ população × 100.000. EMPLASA: Empresa Paulista de Planejamento Metropolitano; IBGE: Instituto Brasileiro de Geografia e Estatística; IGC: Instituto Geográfico e Cartográfico. Fontes do mapa: áreas urbanas do IBGE, limites administrativos do IGC e sub-regiões da EMPLASA da região metropolitana de São Paulo

## Introdução

Todos os anos, milhões de pessoas morrem em decorrência de doenças crônicas não transmissíveis (DCNTs), incluindo neoplasias, diabetes mellitus, doenças cardiovasculares (DCVs) e doenças respiratórias crônicas, muitas das quais são preveníveis e tratáveis. A carga dessas condições é particularmente expressiva em cidades de países do Sul Global, onde as desigualdades sociais e econômicas permanecem substanciais. Segundo a Organização Mundial da Saúde, as DCVs constituem a principal causa de morte no mundo, sendo responsáveis por aproximadamente 17,9 milhões de óbitos anuais.^1^

Pacientes admitidos em hospitais terciários frequentemente apresentam múltiplas comorbidades e necessitam de cuidados de alta complexidade. Após a alta hospitalar, informações de seguimento em longo prazo geralmente não estão disponíveis nos bancos de dados hospitalares, exceto quando os pacientes estão incluídos em protocolos de pesquisa. Lesage et al.^2^ conduziram um estudo de seguimento de 5 anos envolvendo 86 pacientes após a alta hospitalar e reportaram taxa de mortalidade de 33,3%, taxa de readmissão de 21,3% e intervalo médio de 50 dias entre a alta e a readmissão.

Lederman et al.^3^ observaram que os quatro principais grupos de doenças que acometiam os pacientes atendidos em nossa instituição eram doenças do sistema circulatório, do sistema respiratório, do sistema endócrino e neoplasias. Também identificamos duas associações de doenças: uma entre o Capítulo IX (Doenças do Sistema Circulatório) e o Capítulo XVI (Algumas Afecções Originadas no Período Perinatal) da Classificação Estatística Internacional de Doenças e Problemas Relacionados à Saúde, 10ª Revisão (CID-10), e outra entre o Capítulo IX e o Capítulo XVII (Malformações Congênitas, Deformidades e Anomalias Cromossômicas) da CID-10.

O presente estudo teve como objetivo avaliar a distribuição geográfica de 2 grupos de prontuários eletrônicos provenientes de um hospital público terciário: i) pacientes que morreram durante o seguimento e ii) pacientes que permaneceram vivos até o final do período de acompanhamento. A análise concentrou-se em doenças circulatórias, respiratórias, endócrinas e neoplásicas e/ou em seu papel como causas básicas de morte. Nossa hipótese foi de que os achados forneceriam informações relevantes para aprimorar as estratégias de acompanhamento dos pacientes e contribuir com evidências adicionais sobre a distribuição espacial dos principais grupos de doenças nessa população.

## Métodos

### Agrupamento

Com base nos achados de Lederman et al.,^3^ foram definidos dois grupos de doenças para identificar aglomerados de doenças: um representando as principais doenças identificadas durante a primeira consulta hospitalar e outro representando as principais causas básicas de morte. Uma descrição mais detalhada desses grupos está disponível no material suplementar online. A densidade das doenças foi calculada dividindo-se a frequência de casos em cada grupo de doenças pela população da respectiva cidade ou distrito e multiplicando-se o resultado por 100.000 para melhorar a visualização dos dados.

### Dados omitidos

O hospital atende um número substancial de pacientes que realizam apenas procedimentos diagnósticos, como exames de imagem e laboratoriais, sem comparecer a consultas clínicas. Como consequência, esse subgrupo tende a apresentar maior tempo de sobrevida em comparação com pacientes que apresentam um ou mais diagnósticos clínicos.

### Tratamento dos dados

O compartilhamento de dados entre o hospital e o banco público de mortalidade foi regulamentado por meio de um acordo firmado entre ambas as instituições em 28 de dezembro de 2022. Para preservar a confidencialidade dos pacientes, os códigos postais capazes de identificar endereços residenciais foram substituídos pelo código postal mais próximo que não permitisse a identificação do endereço.

### Registros populacionais

Um total de 1.395.063 registros hospitalares coletados entre 2002 e 2017 foi selecionado para vinculação com dados de registro civil do estado de São Paulo, processados e mantidos por uma fundação pública. As estimativas populacionais para o estado de São Paulo em 2017 foram obtidas no repositório do Sistema Estadual de Análise de Dados.^4^

### Variáveis

As variáveis demográficas, hospitalares e relacionadas à mortalidade foram previamente descritas na Seção 2.5 da publicação original.^1^ As variáveis geográficas, incluindo coordenadas de latitude e longitude, foram derivadas dos códigos postais dos endereços dos pacientes.

### Cenário da pesquisa

O estudo foi conduzido em um hospital público terciário universitário utilizando registros de pacientes atendidos entre 1º de janeiro de 2002 e 31 de dezembro de 2017, independentemente de idade ou sexo. A instituição recebe um grande volume de pacientes que necessitam de cuidados relacionados a doenças cardíacas, respiratórias e endócrinas. Devido às exigências de cegamento do periódico, o nome da instituição não pode ser divulgado no presente manuscrito.

### População de pacientes

Aproximadamente 80% dos pacientes atendidos no hospital recebem assistência por meio do Sistema Único de Saúde, enquanto os demais pacientes são cobertos por serviços privados de saúde. Um total de 1.351.070 registros hospitalares foi incluído na análise. Informações sobre o tipo de cobertura em saúde não estavam disponíveis.

### Critérios de inclusão

Todos os registros hospitalares de pacientes entre 1º de janeiro de 2002 e 31 de dezembro de 2018 foram elegíveis para inclusão. Os dados de mortalidade abrangendo o período de 2002 a 2017 foram fornecidos por uma fundação pública. Detalhes adicionais sobre os critérios de inclusão estão disponíveis na Seção 2.8 da publicação anterior.^3^

#### Processo de vinculação de dados

Foi aplicada a metodologia de vinculação determinística,^5^ uma técnica amplamente utilizada por pesquisadores afiliados à fundação (eliminar) responsável pela gestão dos dados de mortalidade.

#### Padronização de variáveis, derivação de variáveis, vinculação de dados e limpeza dos dados

A padronização das variáveis foi realizada para gerar variáveis comparáveis para os procedimentos de vinculação de registros. Métodos de derivação de variáveis foram aplicados para considerar variações ortográficas e melhorar a acurácia da vinculação. Procedimentos de limpeza dos dados foram conduzidos para garantir consistência e confiabilidade entre os bancos de dados.

#### Anonimização

Para garantir a privacidade e a confidencialidade dos pacientes, os responsáveis hospitalares pela proteção de dados exigiram a remoção de todos os identificadores pessoais, incluindo nomes, endereços, números de identificação pública, identificadores dos pacientes e os três últimos dígitos dos códigos postais.

## Análise estatística

**Figura 1:**
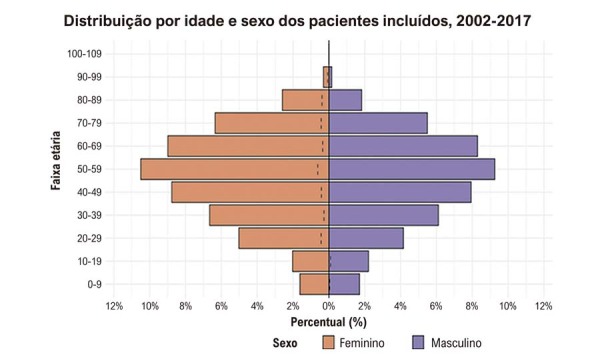
– Distribuição por idade e sexo dos pacientes incluídos entre 2002 e 2017. Fonte: hospital terciário universitário.

Foram realizadas análises descritivas para caracterizar a população do estudo. A pirâmide populacional apresentada na Figura 1 ilustra que as mulheres eram mais velhas do que os homens em todas as faixas etárias. A densidade de mortalidade (Tabela 1) e a densidade de doenças (Tabela 2) foram calculadas utilizando-se a taxa de frequência dividida pela população correspondente do distrito ou cidade.

**Tabela 1:** – Municípios com as maiores taxas de causas básicas de morte

Grupo de doenças	Município	Taxa**
Neoplasias	Vargem Grande Paulista	6,285
	Itapecerica da Serra	2,540
	Itapevi	1,814
	Barueri	1,745
	São Paulo	1,508
Doenças do sistema respiratório	São Paulo	8,386
	Juquitiba	7,092
	Osasco	6,765
	Mairiporã	6,748
	Taboão da Serra	6,440
Doenças endócrinas, nutricionais e metabólicas	Mairiporã	5,624
	Taboão da Serra	4,924
	Vargem Grande Paulista	4,190
	São Paulo	3,478
	Biritiba Mirim	3,400
Algumas afecções originadas no período perinatal	São Paulo	0,009
Doenças do sistema circulatório	São Paulo	58,704
	Osasco	52,713
	Taboão da Serra	51,138
	Mairiporã	39,366
	Carapicuíba	35,223
Malformações congênitas, deformidades e anomalias cromossômicas	Ribeirão Pires	4,330
	Poá	3,780
	Guararema	3,435
	Carapicuíba	3,131
	Embu-Guaçu	3,036

Taxa = Frequência total da doença ÷ população do município × 100.000.

**Tabela 2:** – Municípios com as maiores taxas de comorbidades

Grupo de doenças	Município	Taxa*
Neoplasias	Vargem Grande Paulista	6,285
	Itapecerica da Serra	2,540
	Itapevi	1,814
	Barueri	1,745
	São Paulo	1,508
Doenças do sistema respiratório	Embu das Artes	11,649
	Pirapora do Bom Jesus	11,611
	São Paulo	8,726
	Vargem Grande Paulista	8,380
	Mairiporã	7,873
Doenças endócrinas, nutricionais e metabólicas	São Lourenço da Serra	45,800
	Santa Isabel	41,937
	São Caetano do Sul	39,995
	São Paulo	33,649
	Taboão da Serra	32,198
Algumas afecções originadas no período perinatal	Itapevi	0,453
	Suzano	0,345
	São Paulo	0,035
Doenças do sistema circulatório	Taboão da Serra	257,206
	São Paulo	230,656
	Embu das Artes	189,192
	São Caetano do Sul	169,352
	Osasco	167,299
Malformações congênitas, deformidades e anomalias cromossômicas	Guararema	30,916
	Vargem Grande Paulista	23,046
	Taboão da Serra	21,970
	Santa Isabel	20,968
	Embu das Artes	20,084

Taxa = Frequência total da doença ÷ população do município × 100.000.

### Software

As análises estatísticas foram realizadas utilizando o R versão 4.3.2 (2023-10-31; “Eye Holes”; R Foundation for Statistical Computing, Viena, Áustria), executado na plataforma x86_64-w64-mingw32/x64 (64 bits). Foram utilizados os seguintes pacotes do R: apyramid, data.table, devtools, dplyr, foreign, ggplot2, ggrepel, gridExtra, here, janitor, lubridate, mapview, pacman, paletteer, R.utils, readxl, rgeoda, scales, scattermore, sf, spdep, stringr, tidyr e tidyverse. O ArcGIS versão 10.7 foi utilizado para geração dos mapas.

## Aspectos éticos

O estudo foi aprovado pelo comitê de ética em pesquisa envolvendo seres humanos do hospital terciário (CAAE: 71179723.8.0000.0068; Número do Parecer: 6.618.043).

## Declaração de acesso aos dados

O acesso aos dados foi restrito em conformidade com a Lei Geral de Proteção de Dados (LGPD 13.709/2018). Além disso, a instituição estabeleceu um acordo de confidencialidade com a fundação pública responsável pela gestão dos dados de mortalidade, proibindo a divulgação dos dados a terceiros. As análises de dados foram realizadas em computadores isolados, sem acesso à internet, e protegidos por múltiplas camadas de autenticação. A troca segura e criptografada de dados entre as instituições ocorreu por meio de uma infraestrutura dedicada, disponível apenas por período limitado e acessível exclusivamente mediante credenciais temporárias. Embora os dados brutos não possam ser compartilhados publicamente, os autores estão disponíveis para responder questões específicas de editores ou revisores e estão abertos à inspeção local do banco de dados, se necessário.

## Resultados

Analisamos dados de localização residencial provenientes de aproximadamente 1,3 milhão de registros hospitalares. O banco de dados institucional armazena até os 20 endereços mais recentes de cada paciente, e o endereço registrado mais recentemente foi utilizado nas presentes análises.

Em relação às causas básicas de morte, Vargem Grande Paulista apresentou a maior densidade de neoplasias. São Paulo e Juquitiba apresentaram a maior densidade de doenças do sistema respiratório. Mairiporã e Taboão da Serra apresentaram a maior densidade de doenças endócrinas, nutricionais e metabólicas. São Paulo, Osasco e Taboão da Serra apresentaram a maior densidade de doenças do sistema circulatório. Ribeirão Pires, Poá e Guararema apresentaram a maior densidade de malformações congênitas, deformidades e anomalias cromossômicas. Algumas afecções originadas no período perinatal foram observadas exclusivamente em São Paulo.

Em relação às comorbidades, Vargem Grande Paulista apresentou a maior densidade de neoplasias. Embu das Artes e Pirapora do Bom Jesus apresentaram a maior densidade de doenças do sistema respiratório. São Lourenço da Serra, Santa Isabel e São Caetano do Sul apresentaram a maior densidade de doenças endócrinas, nutricionais e metabólicas. Itapevi e Suzano apresentaram a maior densidade de algumas afecções originadas no período perinatal. Taboão da Serra e São Paulo apresentaram a maior densidade de doenças do sistema circulatório. Guararema, Vargem Grande Paulista e Taboão da Serra apresentaram a maior densidade de malformações congênitas, deformidades e anomalias cromossômicas.

## Discussão

Este estudo relata uma experiência de 9 anos na análise de registros hospitalares de saúde provenientes de um centro público terciário universitário de referência localizado no sudeste do Brasil e especializado em DCVs e respiratórias. O período do estudo abrangeu os anos de 2002 a 2017, durante os quais aproximadamente 1,3 milhão de registros eletrônicos hospitalares foram analisados, e 180.000 óbitos foram identificados durante o seguimento. A presente análise concentrou-se em municípios da região metropolitana de São Paulo.

Informações de mortalidade em longo prazo não estão rotineiramente disponíveis em bancos de dados hospitalares, limitando a capacidade de monitorar os desfechos dos pacientes após a alta hospitalar. Além disso, os hospitais frequentemente não dispõem de informações sobre o intervalo entre o último atendimento hospitalar e o óbito. Ao realizar a vinculação entre dados hospitalares e de mortalidade, nosso estudo contribui para enfrentar essas limitações e pode fornecer informações úteis para avaliação institucional e planejamento em saúde.

Uma publicação recente^6^ relatou que as DCVs e respiratórias foram responsáveis pelo maior número de anos de vida ajustados por incapacidade no Brasil, seguidas pelas doenças respiratórias crônicas e infecciosas, com apenas uma pequena proporção atribuível às mudanças climáticas. Esses achados são particularmente relevantes porque nosso estudo demonstrou que as doenças respiratórias, tanto como diagnósticos quanto como causas básicas de morte, foram mais frequentemente observadas em municípios da região metropolitana de São Paulo caracterizados por grande presença industrial e/ou intenso tráfego veicular. Embora fatores relacionados ao clima não tenham sido avaliados no presente estudo, outra investigação recente^7^ demonstrou os efeitos adversos da poluição ambiental sobre a saúde no Brasil, o que é consistente com a distribuição espacial observada em nossa análise para doenças respiratórias na primeira consulta hospitalar e como causas básicas de morte.

O estudo Spatial Analysis of Risk Areas of Congenital Anomalies in Brazil, 2012-2021^8^ reportou que as anomalias congênitas no Brasil estão predominantemente concentradas na região Nordeste do país, com aglomerados menores identificados no estado de São Paulo. Em contraste, nossos achados demonstraram uma distribuição espacial mais homogênea das anomalias congênitas na região metropolitana de São Paulo, sem evidências de padrões importantes de aglomeração.

Outra investigação, Noncommunicable Diseases Attributed to Low Levels of Physical Activity in Brazil: An Epidemiologic Global Burden of Disease Study,^9^ reportou uma taxa de mortalidade de 293,39 óbitos por 100.000 habitantes no Brasil em 2019 atribuível às DCNTs e às condições associadas aos baixos níveis de atividade física, sendo as DCVs o principal contribuinte.

De forma semelhante, o estudo *The Impact of the Strategic Action Plan to Combat Chronic Noncommunicable Diseases on Hospital Admissions and Deaths From Cardiovascular Diseases in Brazil*^10^ demonstrou reduções nas internações hospitalares relacionadas às DCVs entre 2008 e 2019, mas destacou a persistente carga de mortalidade, particularmente entre populações idosas. Esses achados reforçam a importância do fortalecimento de estratégias de prevenção, sistemas de monitoramento e intervenções direcionadas aos fatores de risco cardiovascular.

### Pontos fortes e limitações do estudo

Os pontos fortes deste estudo incluem o grande volume de registros eletrônicos de saúde analisados ao longo de um período prolongado e a potencial aplicabilidade desses achados para o planejamento local em saúde e a alocação de recursos destinados à oferta de cuidados especializados.

Este estudo também apresenta limitações. Por se tratar de uma experiência institucional de centro único, nossos achados podem não ser representativos da população mais ampla de São Paulo ou de outras regiões do Brasil. Além disso, as análises espaciais foram baseadas nos locais de residência, e não nos locais de trabalho. Também não realizamos estratificação das análises segundo idade ou sexo. Por fim, a categorização das doenças foi baseada em 6 grupos predefinidos derivados de achados institucionais prévios, sendo necessárias investigações futuras para explorar e refinar essas classificações.

## Conclusão

Este estudo apresenta uma análise espacial de registros eletrônicos de saúde provenientes de um hospital público terciário de referência, abrangendo o período de 2002 a 2017, com foco na região metropolitana de São Paulo. A Figura Central apresenta a distribuição geográfica das doenças do sistema circulatório e destaca os municípios com maior densidade de doenças segundo os registros hospitalares.

Os achados demonstraram acentuada heterogeneidade espacial entre municípios e distritos da região metropolitana. Vargem Grande Paulista apresentou a maior densidade de neoplasias tanto como causas básicas de morte quanto como comorbidades. Além disso, municípios com extensas áreas verdes também apresentaram altas densidades de doenças do sistema respiratório. Padrões geográficos também foram identificados para doenças endócrinas, nutricionais e metabólicas, bem como para doenças do sistema circulatório, tanto como causas básicas de morte quanto como comorbidades.

Áreas sem casos registrados devem ser interpretadas com cautela, pois esses achados podem refletir frequências de eventos abaixo dos limiares de visualização cartográfica e/ou menores tamanhos populacionais em regiões geograficamente periféricas.

## Material suplementar

Material suplementar
