# Supplementary material for: Distribuição Espacial de Pacientes na Região Metropolitana de São Paulo Atendidos em um Hospital Público Terciário de Referência Cardiovascular Segundo Doenças dos Sistemas Circulatório, Respiratório, Endócrino e Neoplásico
Source: Arq Bras Cardiol. 2026 Jun 26;123(6):e20260178. [Article in Portuguese] doi: 10.36660/abc.20260178 (PMC13399203; doi:10.36660/abc.20260178)
Supplement: Material suplementar [file 0066-782x-abc-123-6-e20260178-suppl01.pdf]

Attachment - **Literature revision** (750 palavras)

Every year, millions of people lose their lives to chronic noncommunicable diseases - NCDs (neoplasms, diabetes mellitus, cardiovascular and chronic respiratory diseases), which could be prevented and treated, especially in cities of countries of the Global South, where health inequalities and monetary inequities are more evident. According to the World Health Organization (WHO)[5], among NCDs, cardiovascular diseases (CVD) represent the leading cause of death in the world, being responsible for approximately 17.9 million deaths each year (WHO, 2021). CVDs represent a group of diseases of the heart and blood vessels, such as ischemic heart disease, heart failure and cerebrovascular diseases, such as stroke (STEWART et al. 2017)[6]. Diseases of the circulatory system are frequently observed in older adults, especially among adults aged 65 and over (RODGERS et al., 2019)[7]. Several studies (ALMENDRA et al, 2017[8]; HUXLEY and WOODWARD, 2011[9] ; JACKSON et al., 2019[10]; STEWART et al., 2017[6]; SHIBASAKI et al., 2013[11]) point out that CVDs are caused by a variety of complex factors ranging from the genetic characteristics of each individual, lifestyle, as well as the influence of environmental factors (air pollution and extremes of air temperature), socioeconomic factors, and the environment. Although studies indicate a significant reduction in the mortality rate from CVD during the last decades in several countries, due to significant improvements in living conditions and health care, in Brazil, the burden of CVDs is still significant. Approximately 30% (400 thousand) of deaths in the country are related to this cause and it is estimated that 14 million people have CVDs. In São Paulo, the largest Brazilian city, the average annual mortality from CVDs varies between 24 thousand and 25 thousand deaths (PROAIM, 2022). Currently, the city has a population of approximately 11.4 million inhabitants (IBGE, 2023), with a considerable proportion of adults aged 65 or over, an age group that continues to grow. In addition, it presents large socioeconomic and environmental disparities in its territory, which may contribute to the increased risk of mortality and hospital admissions due to CVD. However, there is a lack of scientific evidence that assesses the spatial distribution of CVD in the city, the mortality trend, the areas of influence of the main health services that treat these causes, as well as the analysis of the set of multiple causes of death. Studies that address these issues are essential for planning and forming health measures. The Heart Institute (Incor) is a highly complex hospital widely recognized in São Paulo, is integrated with the Hospital das Clínicas of the Faculty of Medicine of the University of São Paulo and serves thousands of people annually. Incor stands out for its specialization in the treatment, care and prevention of CVD. As a public referral hospital, it serves patients not only from the city of São Paulo, but also from other municipalities in the metropolitan region. Due to this wide activity scope and its importance for the health service, Incor maintains a large database of patient data, providing a good opportunity to improve our understanding of CVD mortality in the metropolitan area of São Paulo. Therefore, this study aims to explore the Incor database in order to assess the hospital's area of influence, identify the spatial pattern of CVD mortality recorded by the hospital, verify the social mobility of patients treated by the

hospital, and evaluate the multiple causes of death and certain outcomes associated with CVD. Objectives The main objective of this research project was to explore and evaluate the hospital`s database using spatial and statistical analyses in order to contribute to a better understanding of CVD in São Paulo.

Patients admitted to tertiary hospitals often carriers of multiple comorbidities and have complex resolution. After discharge, their follow-up is rarely recorded in the hospital's database except when the patient is followed by a research protocol. In this case, data from Lesage et al[5],with a 5-year follow-up and with a small sample of 86 patients after hospital discharge, found 33.3% of mortality, 21.3% of readmission , with an average time between discharge and readmission of 50 days.

In the same direction, McCarthy CP et al[6], also with an exceedingly small sample of patients, involved 359 patients admitted with myocardial infarction and found that 10% of them died in the hospital and 19.7% survived (at home) for one year.
In addition, when assessing the total sample of patients, 23.2% mortality from all causes and 69.2% readmission from all causes identified. When they analyzed the main combined events, they found 34.9% mortality, myocardial infarction, or stroke.

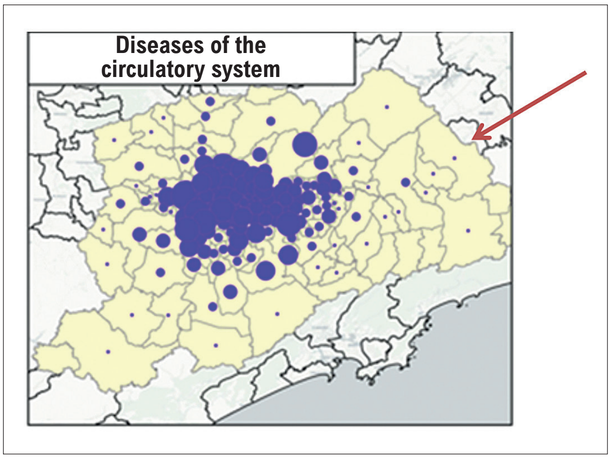

Figure 2 – *Diseases of the circulatory system as underlying causes of death. In addition to the high concentration of deaths related to diseases of the circulatory system in the central region of the map, Guarulhos (A) also showed a notably high concentration of cases.*

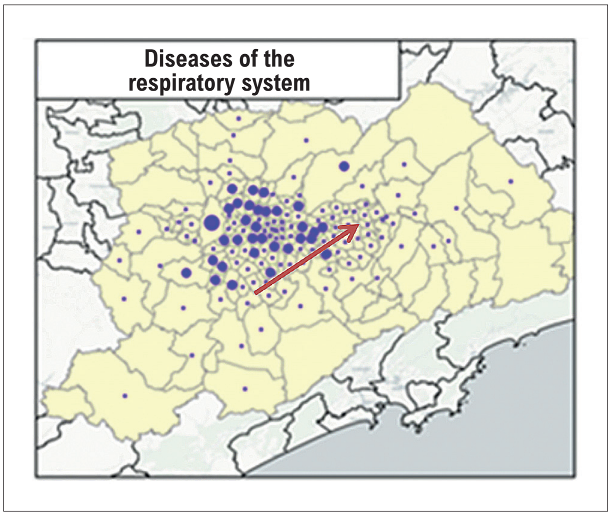

Figure 3 – *Diseases of the respiratory system as underlying causes of death. Osasco (A) showed a high concentration of diseases of the respiratory system.*

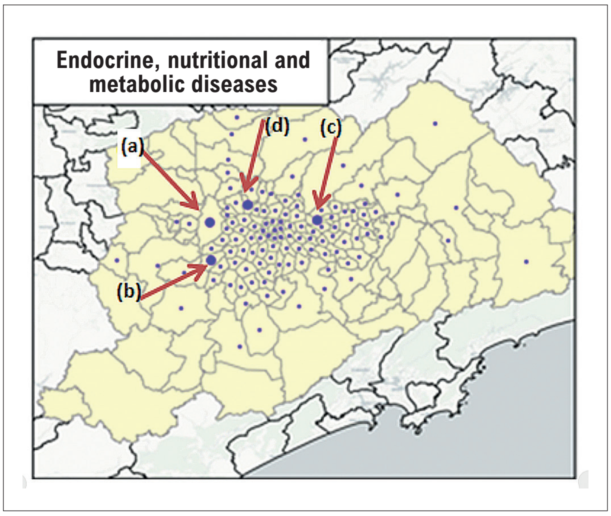

Figure 4 – *Endocrine and metabolic diseases as underlying causes of death. Osasco (A), Embu das Artes (B), Itaquaquecetuba (C), and the northeastern region of São Paulo (D) showed high concentrations of deaths related to endocrine and metabolic diseases. A more homogeneous spatial distribution was observed for neoplasms, certain conditions originating in the perinatal period, and congenital malformations, deformations, and chromosomal abnormalities.*

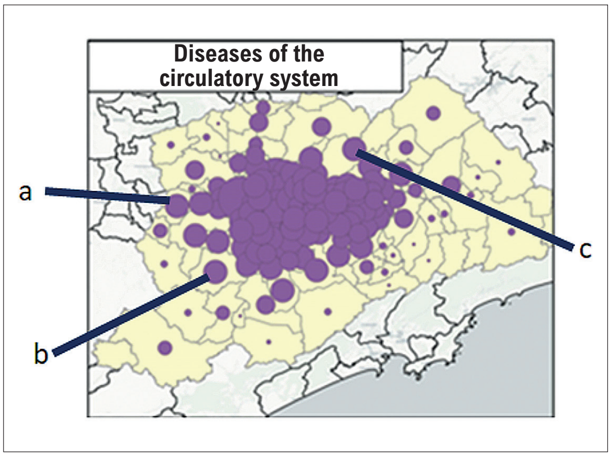

Figure 5 – *Diagnoses of diseases of the circulatory system. Cotia (A), Itapecerica da Serra (B), and Itaquaquecetuba (C) were the peripheral cities/districts with the highest number of cases.*

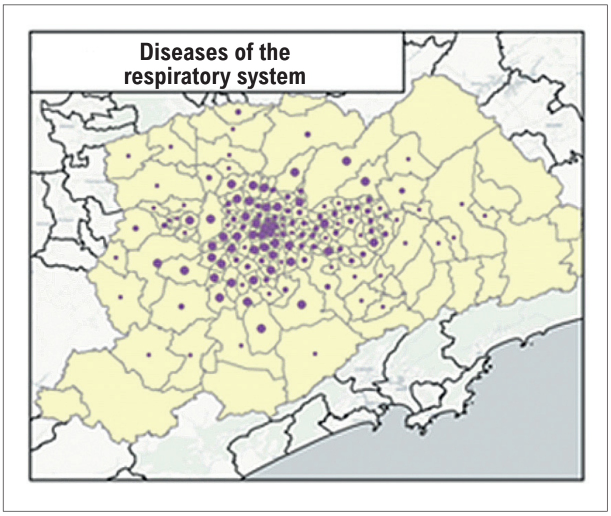

Figure 6 – *Diagnoses of diseases of the respiratory system. Cities/districts with the highest concentrations of diseases of the respiratory system.*

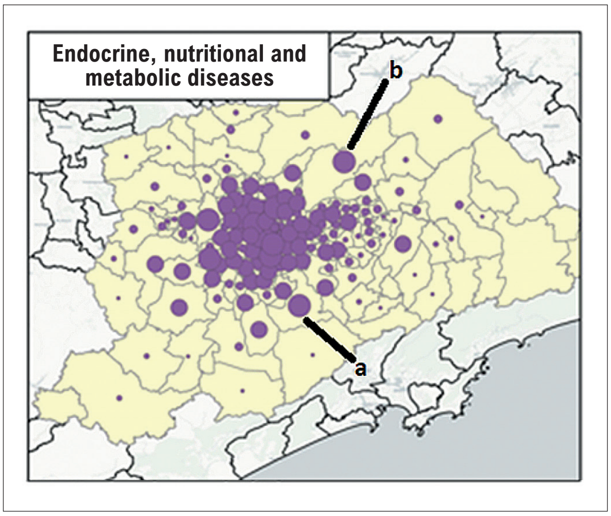

Figure 7 – *Diagnoses of endocrine, nutritional and metabolic diseases. Cities/districts with the highest concentrations of endocrine, nutritional, and metabolic diseases: Mogi das Cruzes (A) and Guarulhos (B).*

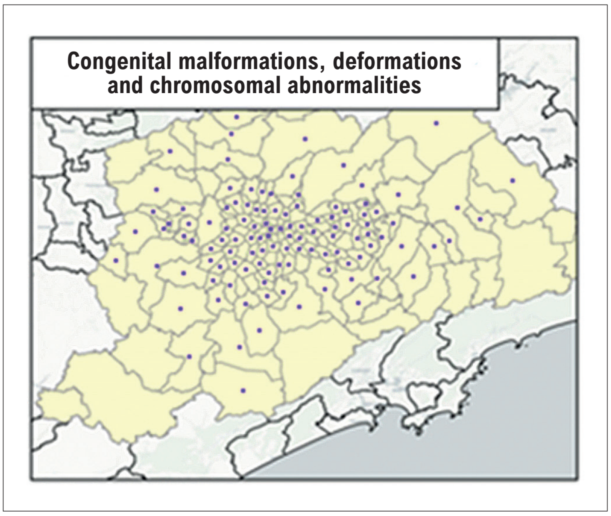

Figure 8 – *Diagnoses of congenital malformations, deformations and chromosomal abnormalities. Cities/districts showed a homogeneous spatial distribution of congenital malformations, deformations, and chromosomal abnormalities.*
