# Supplementary material for: Distribuição Espacial de Pacientes na Região Metropolitana de São Paulo Atendidos em um Hospital Público Terciário de Referência Cardiovascular Segundo Doenças dos Sistemas Circulatório, Respiratório, Endócrino e Neoplásico
Source: Arq Bras Cardiol. 2026 Jun 26;123(6):e20260178. [Article in Portuguese] doi: 10.36660/abc.20260178 (PMC13399203; doi:10.36660/abc.20260178)
Supplement: Translation

# Translation: Spatial Distribution of Patients in the Metropolitan Area of São Paulo Attending a Public Tertiary Cardiovascular Referral Hospital According to Diseases of the Circulatory, Respiratory, Endocrine, and Neoplastic Systems

**DOI:** 10.36660/abc.20260178i

**Authors:** Carlos Lederman, Sara Lopes de Moraes, Antonio Carlos Pedroso de Lima, Lucia Pereira Barroso, Lilian Cristina Correia Morais, Magaly de Losso Perdigão, Rosa Maria Vieira de Freitas, Monica La Porte Teixeira, Bernadette Cunha Waldvogel, Marco Antonio Gutierrez, Ligia Vizeu Barrozo, Alfredo José Mansur

**Affiliations:** 1 Brazil Faculdade de Medicina da Universidade de São Paulo (FMUSP), São Paulo, SP – Brazil; 2 Brazil Universidade de São Paulo – Faculdade de Filosofia, Letras e Ciências Humanas, São Paulo, SP – Brazil; 3 Brazil Universidade de São Paulo – Instituto de Matemática e Estatística, São Paulo, SP – Brazil; 4 Brazil Fundação Sistema Estadual de Análise de Dados – Projeções Populacionais, São Paulo, SP – Brazil; 5 Brazil Instituto do Coração do Hospital das Clínicas da Faculdade de Medicina da Universidade de São Paulo, São Paulo, SP – Brazil

**Keywords:** International Classification of Diseases, Comorbidity, Cause of Death, Mortality, Noncommunicable Diseases

## Abstract

**Background:**

The spatial distribution of patients according to clinical characteristics, using geoprocessing techniques, enables the identification of disease clusters within a studied population. This approach supports the development of targeted interventions directed toward specific risk groups.

**Objectives:**

To investigate geographical clusters of diseases of interest among patients attending a public tertiary hospital and to characterize their spatial distribution across the metropolitan area of São Paulo.

**Methods:**

Based on the results of a previous study, two disease groups were defined: one representing the main diseases identified at the first hospital visit and another representing the main underlying causes of death. A detailed description is provided in the online supplementary material. Disease density was calculated by dividing the frequency of cases within each disease group by the population of each city/district and multiplying the result by 100,000 to enhance data visualization. Spatial data were subsequently analyzed and mapped to illustrate the spatial patterns and relationships identified in the study.

**Results:**

Osasco and Taboão da Serra showed the highest density of underlying causes of death related to diseases of the circulatory system. Additionally, 12 cities/districts located at the borders of the study area showed no recorded neoplastic diseases.

**Conclusions:**

Vargem Grande Paulista showed the highest case density for neoplasms as underlying causes of death and comorbidities. Notably, Embu das Artes, a city recognized for its extensive green areas, showed the highest density of diseases of the respiratory system.

## Introduction

Every year, millions of people die from chronic noncommunicable diseases (NCDs), including neoplasms, diabetes mellitus, cardiovascular diseases (CVDs), and chronic respiratory diseases, many of which are preventable and treatable. The burden of these conditions is particularly pronounced in cities across countries of the Global South, where social and economic inequalities remain substantial. According to the World Health Organization, CVD are the leading cause of death worldwide, accounting for approximately 17.9 million deaths annually.^1^

Patients admitted to tertiary hospitals frequently present with multiple comorbidities and require highly complex care. Following hospital discharge, long-term follow-up information is often unavailable in hospital databases unless patients are enrolled in research protocols. Lesage et al.^2^ conducted a 5-year follow-up study involving 86 patients after hospital discharge and reported a mortality rate of 33.3%, a readmission rate of 21.3%, and an average interval of 50 days between discharge and readmission.

Lederman et al.^3^ observed that the four major disease groups affecting patients treated at our institution were diseases of the circulatory system, respiratory system, endocrine system, and neoplasms. We also identified two disease associations: one between ICD-10 Chapter IX (Diseases of the Circulatory System) and Chapter XVI (Certain Conditions Originating in the Perinatal Period), and another between ICD-10 Chapter IX and Chapter XVII (Congenital Malformations, Deformations and Chromosomal Abnormalities).

The present study aimed to evaluate the geographic distribution of 2 groups of electronic health records from a tertiary public hospital: i) patients who died during follow-up and ii) patients who remained alive until the end of the follow-up period. The analysis focused on circulatory, respiratory, endocrine, and neoplastic diseases and/or their role as underlying causes of death. We hypothesized that the findings would provide valuable information to improve patient follow-up strategies and contribute additional evidence regarding the spatial distribution of major disease groups in this population.

## Methods

### Clustering

Based in the findings of Lederman et al.,^3^ two disease groups were defined to identify disease clusters: one representing the main diseases identified during the first hospital visit and another representing the main underlying causes of death. A more detailed description of these groups is available in the online supplementary material. Disease density was calculated by dividing the frequency of cases within each disease group by the population of the corresponding city or district and multiplying the result by 100,000 to improve data visualization.

### Omitted data

The hospital serves a substantial number of patients who undergo only diagnostic procedures, such as imaging and laboratory testing, without attending clinical appointments. As a consequence, this subgroup tends to exhibit longer survival times than patients presenting with one or more clinical diagnoses.

### Data handling

Data sharing between the hospital and the public mortality database was regulated through an agreement signed by both institutions on December 28, 2022. To preserve patient confidentiality, postal codes capable of identifying residential addresses were replaced with the nearest postal code that did not permit address identification.

### Population records

A total of 1,395,063 hospital records collected between 2002 and 2017 were selected for linkage with civil registry data from the state of São Paulo, processed and maintained by a public foundation. Population estimates for the state of São Paulo in 2017 were obtained from the repository of the State Data Analysis System.^4^

### Variables

Demographic, hospital-related, and mortality-related variables were previously described in Section 2.5 of the original publication.^1^ Geographic variables, including latitude and longitude coordinates, were derived from patients’ postal address codes.

### Research setting

The study was conducted at a public tertiary academic hospital using records from patients treated between January 1, 2002, and December 31, 2017, regardless of age or sex. The institution receives a large volume of patients requiring care for cardiac, respiratory, and endocrine diseases. Due to journal blinding requirements, the institution’s name cannot be disclosed in the present manuscript.

### Patient population

Approximately 80% of patients treated at the hospital receive care through the Brazilian Unified Health System, whereas the remaining patients are covered by private health care services. A total of 1,351,070 hospital records were included in the analysis. Information regarding health care coverage type was not available.

### Inclusion criteria

All hospital patient records from January 1, 2002, through December 31, 2018, were eligible for inclusion. Mortality data covering the period from 2002 to 2017 were provided by a public foundation. Additional details regarding inclusion criteria are available in Section 2.8 of the previous publication.^3^

#### Data linkage process

Deterministic linkage methodology was applied,^5^ a technique extensively used by researchers affiliated with the public foundation responsible for mortality data management.

#### Variable standardization, variable derivation, data linkage, and data cleaning

Variable standardization was performed to generate comparable variables for record matching procedures. Variable derivation methods were applied to account for orthographic variations and improve linkage accuracy. Data cleaning procedures were conducted to ensure consistency and reliability across databases.

#### Anonymization

To ensure patient privacy and confidentiality, hospital officers responsible for data protection required the removal of all personal identifiers, including names, addresses, public identification numbers, patient identifiers, and the final three digits of postal codes.

## Statistical analysis

Descriptive analyses were performed to characterize the study population. The population pyramid presented in Figure 1 illustrates that women were older than men across all age groups. Mortality density (Table 1) and disease density (Table 2) were calculated using the frequency rate divided by the corresponding district or city population.

**Figure 1:** – Age and sex distribution of patients included between 2002 and 2017. Source: tertiary academic hospital.

**Table 1:** – Cities with the highest rates of underlying causes of death

Disease group	City	Rate**
Neoplasms	Vargem Grande Paulista	6.285
	Itapecerica da Serra	2.540
	Itapevi	1.814
	Barueri	1.745
	São Paulo	1.508
Diseases of the respiratory system	São Paulo	8.386
	Juquitiba	7.092
	Osasco	6.765
	Mairiporã	6.748
	Taboão da Serra	6.440
Endocrine, nutritional and metabolic diseases	Mairiporã	5.624
	Taboão da Serra	4.924
	Vargem Grande Paulista	4.190
	São Paulo	3.478
	Biritiba Mirim	3.400
Certain conditions originating in the perinatal period	São Paulo	0.009
Diseases of the circulatory system	São Paulo	58.704
	Osasco	52.713
	Taboão da Serra	51.138
	Mairiporã	39.366
	Carapicuíba	35.223
Congenital malformations, deformations, and chromosomal abnormalities	Ribeirão Pires	4.330
	Poá	3.780
	Guararema	3.435
	Carapicuíba	3.131
	Embu-Guaçu	3.036

Rate = Total disease frequency ÷ city population × 100,000.

**Table 2:** – Cities with the highest rates of comorbidities

Disease group	City	Rate**
Neoplasms	Vargem Grande Paulista	6.285
	Itapecerica da Serra	2.540
	Itapevi	1.814
	Barueri	1.745
	São Paulo	1.508
Diseases of the respiratory system	Embu das Artes	11.649
	Pirapora do Bom Jesus	11.611
	São Paulo	8.726
	Vargem Grande Paulista	8.380
	Mairiporã	7.873
Endocrine, nutritional and metabolic diseases	São Lourenço da Serra	45.800
	Santa Isabel	41.937
	São Caetano do Sul	39.995
	São Paulo	33.649
	Taboão da Serra	32.198
Certain conditions originating in the perinatal period	Itapevi	0.453
	Suzano	0.345
	São Paulo	0.035
Diseases of the circulatory system	Taboão da Serra	257.206
	São Paulo	230.656
	Embu das Artes	189.192
	São Caetano do Sul	169.352
	Osasco	167.299
Congenital malformations, deformations and chromosomal abnormalities	Guararema	30.916
	Vargem Grande Paulista	23.046
	Taboão da Serra	21.970
	Santa Isabel	20.968
	Embu das Artes	20.084

Rate = Total disease frequency ÷ city population × 100,000.

### Software

Statistical analyses were performed using R version 4.3.2 (2023-10-31; “Eye Holes”; R Foundation for Statistical Computing, Vienna, Austria) running on the x86_64-w64-mingw32/x64 (64-bit) platform. The following R packages were used: apyramid, data.table, devtools, dplyr, foreign, ggplot2, ggrepel, gridExtra, here, janitor, lubridate, mapview, pacman, paletteer, R.utils, readxl, rgeoda, scales, scattermore, sf, spdep, stringr, tidyr, and tidyverse. ArcGIS version 10.7 was used for map generation.

## Ethical aspects

The study was approved by the human research ethics committee of the tertiary hospital (CAAE: 71179723.8.0000.0068; Approval Number: 6.618.043).

## Data access statement

Data access was restricted in accordance with the Brazilian Data Protection Law (LGPD 13.709/2018). Furthermore, the institution established a nondisclosure agreement with the public foundation responsible for mortality data management, prohibiting disclosure of data to third parties. Data analyses were conducted on isolated computers without internet access and protected through multiple authentication layers. Secure encrypted data exchange between institutions occurred through a dedicated infrastructure available only for a limited period and accessible exclusively through temporary credentials. Although raw data cannot be publicly shared, the authors are available to address specific questions from editors or reviewers and are open to local database inspection if required.

## Results

We analyzed residential location data from approximately 1.3 million hospital health records. The institutional database stores up to the 20 most recent addresses for each patient, and the most recent registered address was used for the present analyses.

Regarding underlying causes of death, Vargem Grande Paulista showed the highest density of neoplasms. São Paulo and Juquitiba showed the highest density of diseases of the respiratory system. Mairiporã and Taboão da Serra showed the highest density of endocrine, nutritional, and metabolic diseases. São Paulo, Osasco, and Taboão da Serra showed the highest density of diseases of the circulatory system. Ribeirão Pires, Poá, and Guararema showed the highest density of congenital malformations, deformations, and chromosomal abnormalities. Certain conditions originating in the perinatal period were observed exclusively in São Paulo.

Regarding comorbidities, Vargem Grande Paulista showed the highest density of neoplasms. Embu das Artes and Pirapora do Bom Jesus showed the highest density of diseases of the respiratory system. São Lourenço da Serra, Santa Isabel, and São Caetano do Sul showed the highest density of endocrine, nutritional, and metabolic diseases. Itapevi and Suzano showed the highest density of certain conditions originating in the perinatal period. Taboão da Serra and São Paulo showed the highest density of diseases of the circulatory system. Guararema, Vargem Grande Paulista, and Taboão da Serra showed the highest density of congenital malformations, deformations, and chromosomal abnormalities.

## Discussion

This study reports a 9-year experience in the analysis of hospital health records from a public tertiary academic referral center located in southeastern Brazil and specialized in cardiovascular and respiratory diseases. The study period covered 2002 to 2017, during which approximately 1.3 million electronic hospital health records were analyzed, and 180,000 deaths were identified during follow-up. The present analysis focused on cities within the metropolitan area of São Paulo.

Long-term mortality information is not routinely available in hospital databases, limiting the ability to monitor patient outcomes after hospital discharge. In addition, hospitals often lack information regarding the interval between the last hospital encounter and death. By linking hospital and mortality data, our study contributes to addressing these limitations and may provide useful information for institutional assessment and health care planning.

A recent publication^6^ reported that cardiovascular and respiratory diseases accounted for the greatest disability-adjusted life-years in Brazil, followed by chronic and infectious respiratory diseases, with only a small proportion attributable to climate change. These findings are particularly relevant because our study showed that respiratory diseases, both as diagnoses and underlying causes of death, were more frequently observed in cities within the metropolitan area of São Paulo characterized by a large industrial presence and/or intense vehicular traffic. Although climate-related factors were not evaluated in the present study, another recent investigation^7^ demonstrated the adverse health effects of environmental pollution in Brazil, which is consistent with the spatial distribution observed in our analysis for respiratory diseases at the first hospital visit and as underlying causes of death.

The study Spatial Analysis of Risk Areas of Congenital Anomalies in Brazil, 2012-2021^8^ reported that congenital anomalies in Brazil are predominantly concentrated in the northeastern region of the country, with smaller clusters identified in the state of São Paulo. In contrast, our findings demonstrated a more homogeneous spatial distribution of congenital anomalies across the metropolitan area of São Paulo, without evidence of major clustering patterns.

Another investigation, Noncommunicable Diseases Attributed to Low Levels of Physical Activity in Brazil: An Epidemiologic Global Burden of Disease Study,^9^ reported a mortality rate of 293.39 deaths per 100,000 inhabitants in Brazil in 2019 attributable to NCDs and conditions associated with low physical activity, with CVDs representing the most prominent contributor.

Similarly, the study The Impact of the Strategic Action Plan to Combat Chronic Noncommunicable Diseases on Hospital Admissions and Deaths From Cardiovascular The impact of the strategic action plan to combat chronic noncommunicable diseases on Hospital Admissions and deaths from cardiovascular diseases in Brazil,10 demonstrated reductions in hospital admissions related to CVD between 2008 and 2019 but highlighted the persistent burden of mortality, particularly among older populations. These findings reinforce the importance of strengthening prevention strategies, monitoring systems, and interventions targeting cardiovascular risk factors.

### Strengths and limitations of study

Strengths of this study include the large volume of electronic health records analyzed over an extended period and the potential applicability of these findings to local health care planning and resource allocation for specialized care delivery.

This study also has limitations. As a single-center institutional experience, our findings may not be representative of the broader population of São Paulo or other regions of Brazil. In addition, spatial analyses were based on residential locations rather than workplace locations. We also did not stratify analyses according to age or sex. Finally, disease categorization was based on 6 predefined groups derived from previous institutional findings, and future investigations are warranted to further explore and refine these classifications.

## Conclusion

This study presents a spatial analysis of electronic health records from a tertiary public referral hospital covering the period from 2002 to 2017, focusing on the metropolitan area of São Paulo. Central Illustration displays the geographic distribution of diseases of the circulatory system and highlights cities with the highest disease density according to hospital records.

The findings demonstrated marked spatial heterogeneity across cities and districts within the metropolitan region. Vargem Grande Paulista showed the highest density of neoplasms both as underlying causes of death and as comorbidities. In addition, cities with substantial green areas also showed high densities of diseases of the respiratory system. Geographic patterns were also identified for endocrine, nutritional, and metabolic diseases, as well as diseases of the circulatory system, both as underlying causes of death and comorbidities.

Areas with zero recorded cases should be interpreted cautiously, as these findings may reflect event frequencies below cartographic visualization thresholds and/or smaller population sizes in geographically peripheral regions.

## Supplemental Materials

Supplementary material
